# An exploratory study of urinary proteome in trigeminal neuralgia

**DOI:** 10.3389/fmolb.2025.1618014

**Published:** 2025-10-30

**Authors:** Lilong Wei, Haitong Wang, Yun Zhou, Jianqiang Wu, Yongtong Cao, Yuliang Zhan, Youhe Gao

**Affiliations:** ^1^ Clinical Laboratory, China-Japan Friendship Hospital, Beijing, China; ^2^ Gene Engineering Drug and Biotechnology Beijing Key Laboratory, College of Life Sciences, Beijing Normal University, Beijing, China; ^3^ Institute of Clinical Medicine, National Infrastructure for Translational Medicine, State Key Laboratory of Complex Severe and Rare Diseases, Peking Union Medical College Hospital, Chinese Academy of Medical Sciences & Peking Union Medical College, Beijing, China

**Keywords:** neuropathy, disease markers, urinary proteomics, trigeminal neuralgia, immune reaction

## Abstract

**Background:**

Trigeminal neuralgia (TN) is a neurological disorder characterized by severe pain and a complex pathogenesis. This condition seriously affects patients’ quality of life. The analysis of urinary proteomics has shown great potential in disease research.

**Objective:**

The aim of this study is to explore the diagnostic and therapeutic value of urine proteomics in TN.

**Method:**

The urine of 10 cases of TN and 11 healthy individuals were studied using liquid chromatography-mass spectrometry (LC-MS) proteomics technology. Group analysis and one to many individual analysis strategies were used in quantitative analysis.

**Result:**

A total of 2,620 proteins were identified in the group analysis, and 1,883 of them were quantifiable. Most specimens in each group were distinguishable through clustering analysis of the urinary proteome. Significant enrichment was observed in biological processes such as immune response-regulating signaling pathway, regulation of complement activation, natural killer cell inhibitory signaling pathway, and cytoplasmic translation, as well as in KEGG pathways including antigen processing and presentation, and complement and coagulation cascades. Individual analysis revealed a higher number of significantly different proteins between patients, with seven proteins identified in at least nine patients.

**Conclusion:**

The urinary proteome provided molecular characteristics of urinary proteins in patients. It described the changes that occur in the patient’s body. The strong enrichment of immune response pathways may be linked to disease onset, suggesting new avenues for immune-based treatments, and the significant differential proteins could become potential disease markers for diagnosis and treatment evaluation.

## 1 Introduction

Trigeminal neuralgia (TN) is a type of neurological pain characterized by severe facial pain, usually unilateral in nature. The pain is typically paroxysmal, stabbing, or electric shock-like, lasting from a few seconds to a few minutes, and occurs at a relatively high frequency (Carnevale and Knopman, 2023). TN often affects the condition of basic human psychological, physiological and social needs and activities. Although TN symptoms are well-documented, there is currently no gold standard or specific biomarker for diagnosis (Finnerup et al., 2021). The development of proteomics technology tends to comprehensively discover disease markers (Gallien and Domon, 2015; Dayon et al., 2022), urine, as the most non-invasive source of disease markers, is widely used in clinical research (Shao et al., 2011; Youhe, 2013), at present, there is no search for the use of urinary proteomics technology to study related diseases. This study is the first attempt to use urinary proteomics to study TN, aiming to explore the characteristics of urinary proteomics in patients with TN and to find urine nerve injury related markers.

## 2 Materials and methods

### 2.1 Experimental design and urine sample collection

This study was approved by the Ethics Committee of China-Japan Friendship Hospital (Approval No. 2023-ky-126), and participants were from patients and healthy physical examinees of China-Japan Friendship Hospital. Patients were diagnosed of TN without tumor or kidney diseases. Urine proteomics technology was used to analyze 11 healthy individuals (7 males, 4 females; age range 32–71, mean 51, standard deviation 11) and 10 patients with trigeminal neuralgia (TN) (6 males, 4 females; age range 38–68, mean 54, standard deviation 10). All samples were collected immediately after clinical testing and stored in a −80 °C ultra-low temperature freezer.

### 2.2 Processing of urine samples

Urine proteins were precipitated using ethanol (Shao et al., 2019), then protein from each sample were digested with the filter-aided sample preparation (FASP) method (Wisniewski et al., 2009). Specifically, as follows:The collected urine samples were centrifuged at 12,000 *g* for 30 min at 4 °C, and the supernatant was transferred to a 50 mL centrifugal tube. Dithiothreitol solution (DTT, Sigma) was then added to a final concentration of 20 mM, and the mixture was shaken and incubated in a water bath at 37 °C for 1 h before being cooled to room temperature. Iodoacetamide (IAA, Sigma) was added to a final concentration of 50 mM, and the mixture was shaken and reacted in the dark at room temperature for 40 min. Six times the volume of pre-cooled absolute ethanol was added, and the mixture was homogeneously mixed and precipitated at −20 °C for 24 h. On the second day, the mixture was centrifuged at 4 °C, 12,000×g for 30 min, and the supernatant was discarded. The protein precipitate was resuspended in lysis buffer (containing 8 mol/L urea, 2 mol/L thiourea, 25 mmol/L dithiothreitol, 50 mmol/L Tris). After centrifugation at 12,000 *g* for 30 min at 4 °C, the supernatant was placed in a new EP tube. The protein concentration was measured by the Bradford method. Urine protein digestion: 100 μg urine protein sample was added to the filter membrane of 10 kDa ultrafiltration tube (Pall, Port Washington, NY, United States) and placed in an EP tube, and 25 mmol/L NH_4_HCO_3_ solution was added to make the total volume 200 μL. Then the membrane washing operation was carried out: ① 200 μL UA solution (8 mol/L urea, 0.1 mol/L Tris-HCl, pH 8.5) was added and centrifuged and washed twice at 14,000×g 5 min 18 °C; ② Loading: the sample was added and centrifuged at 14,000 *g* 40 min 18 °C; ③ 200 μL UA solution was added and centrifuged at 14,000 *g* for 40 min at 18 °C, repeated 2 times; ④ 25 mmol/L NH_4_HCO_3_ solution was added and centrifuged at 14,000 *g* 40 min 18 °C for 3–4 times; ⑤ Trypsin (Trypsin Gold, Promega, Fitchburg, WI, United States) was added at a ratio of 1:50 for digestion, and the water bath was kept at 37 °C for 12–16 h. The next day, the peptide segment was collected by centrifugation at 13,000 *g* 30 min 4 °C, and passed through the HLB column (Waters, Milford, MA, United States) for desalting. The eluent was vacuum dried at 4 °C for approximately 1.5 h. The peptide segments were then collected and stored at −80 °C.

### 2.3 LC-MS/MS tandem mass spectrometry analysis

The digested samples were dissolved in 0.1% formic acid and quantified using the BCA kit. Equivalent peptide amount was loaded. The peptide concentration was diluted to 0.5 μg/μL. Four μL of each sample was taken to prepare the mixed polypeptide sample, and the separation was performed using a high pH reversed phase peptide separation kit (Thermo Fisher Scientific) according to the instructions. Ten fractions were collected by centrifugation, and after drying using a vacuum dryer, they were resuspended in 0.1% formic acid. For analysis, 1 μg of peptides from each sample was taken, and mass spectrometry analysis and data acquisition were performed using an EASY-nLC1200 chromatography system (Thermo Fisher Scientific, United States) and an Orbitrap Fusion Lumos Tribrid mass spectrometer (Thermo Fisher Scientific, United States). In order to generate the spectral library, the separated 10 Fractions were analyzed by mass spectrometry in Data Dependent Acquisition (DDA) mode. The mass spectrometry data were collected in high sensitivity mode. A complete mass spectrometry scan was obtained in the range of 350–1500 m/z with a resolution setting of 60,000. Individual samples were analyzed using the Data Independent Acquisition (DIA) mode. DIA acquisition was performed using a DIA method with 36 windows. The iRT reagent (Biognosys, Switzerland) was added at a sample: iRT volume ratio of 10:1 to calibrate the retention time of the extracted peptide peaks. After every 8 samples, a single DIA analysis of pooled peptides was performed as quality control (Pan et al., 2022). All experimental specimens were completed in the same batch of experiments.

### 2.4 Database searching and label-free DIA quantification

The raw data (RAW files) acquired from LC-MS/MS were imported into Proteome Discoverer (version 2.1, Thermo Scientific) and searched against the SwissProt database (taxonomy: Homo; containing 20,346 sequences). The iRT sequences were added to the database for comparison. The search results were then imported into Spectronaut Pulsar (Biognosys AG, Switzerland) for processing and analysis. The abundance of peptides was calculated by summing the peak areas of their respective fragment ions in MS_2_. The protein intensity was calculated by summing the abundances of their respective peptides.

### 2.5 Data analysis

Each sample was performed in triplicate, and all three values were used for statistical analysis. The identified proteins were compared, and differentially expressed proteins were screened. The loose screening criteria for differentially expressed proteins were: fold change (FC) ≥ 1.5 or ≤0.67, and adjusted p-value <0.05 by two-tailed unpaired t-test analysis. The strict screening criteria for differentially expressed proteins were: FC ≥ 2 or ≤0.5, and adjusted p-value <0.01 by two-tailed unpaired t-test analysis. To further evaluate the reliability of differentially expressed proteins, the probability of random generation of differentially expressed proteins was calculated by random grouping using the average value of each protein. All samples were randomly grouped to identify differentially expressed proteins, then the ratio of the differential proteins generated under different random methods to that in the experiment were calculated to evaluate the reliability of the results. Functional enrichment analysis of the screened differentially expressed proteins was performed using the Wukong platform (https://www.omicsolution.org/wkomic/main/), Uniprot website (https://www.uniprot.org/) and DAVID database (https://david.ncifcrf.gov/). The reported literature was retrieved from the Pubmed database (https://pubmed.ncbi.nlm.nih.gov) for functional analysis of differentially expressed proteins.

## 3 Experiment results

### 3.1 Urine proteome mass spectrometry identification and analysis

**FIGURE 1:**
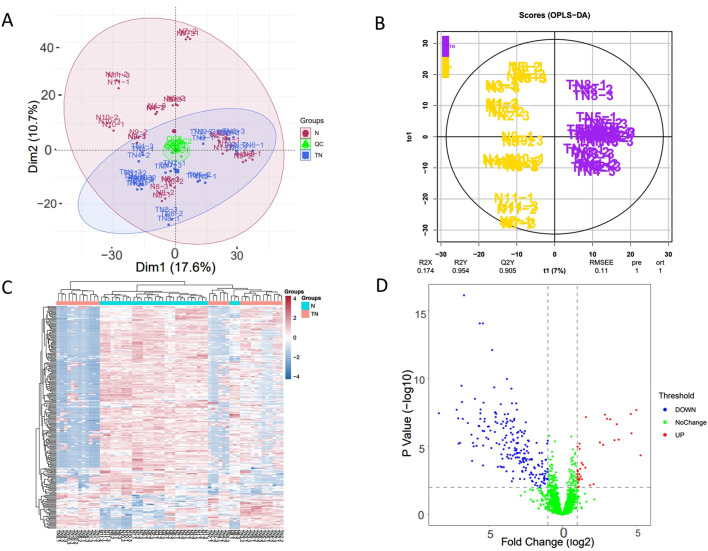
Visualized analysis of identified proteins in in patients with TN and healthy controls. TN, patients with Trigeminal neuralgia; N, healthy controls; QC, Quality control samples. **(A)** principal component analysis (PCA), the data includes three repeated mass spectrometry detections of the same specimen; **(B)** orthogonal partial least squares discrimination analysis (OPLS-DA) based on orthogonal signal correction; **(C)** Hierarchical Cluster Analysis (HCA), significant differential proteins (FC ≥ 1.5 or ≤0.67, and adjusted p-value <0.05); **(D)** Volcano plot of urinary proteome.

**FIGURE 2:**
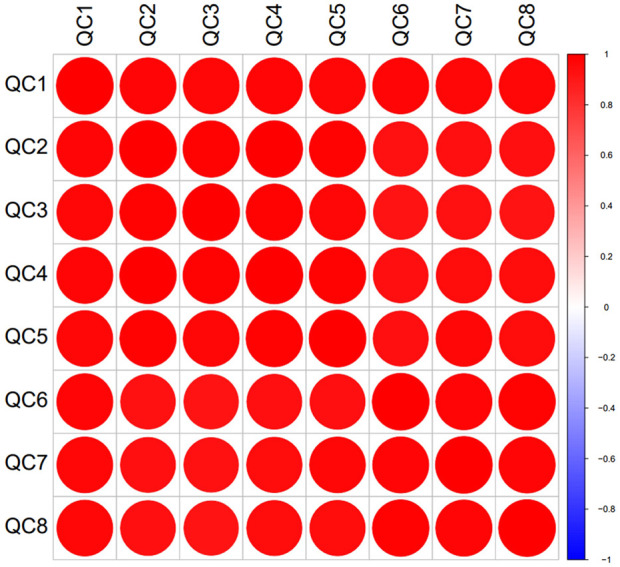
Correlation analysis of quality control samples.

In the experiment, urine samples from 11 healthy individuals (N) and 10 patients with trigeminal neuralgia (TN) were studied. There was no significant difference in gender and age between the two groups of people (Supplementary Material). A total of 2,620 proteins with specific peptide were identified by LC-MS/MS, with protein level FDR<1%. 1,883 proteins (missing values less than 50% in quality control samples) could be used for quantitative analysis. Figures 1A,B showed the PCA and OPLS-DA results for the two sample groups. Most patients with TN were clearly separated from healthy controls, and many significantly different proteins were identified between the two groups based on the volcano plot (Figure 1D). The correlation analysis of QC specimen mass spectrometry data (Figure 2) demonstrated that the instrument remained stable throughout the experimental process. Each specimen was analyzed three times by mass spectrometry, and after data processing, no significant differences were found within the replicates (Figures 1A,C).

### 3.2 Differential proteome analysis of whole urine proteome

#### 3.2.1 Quantitative analysis of differential proteins

The experimental samples from the two groups were compared, and the number of differentially expressed proteins was shown in Table 1. The significance levels for these proteins under two screening conditions were 0.677 and 0.871, respectively. Based on the strict screening criteria for differentially expressed proteins: FC ≥ 2 or ≤0.5, and adjusted p-value <0.01 by two-tailed unpaired t-test analysis, 238 proteins were found significantly changed (Supplementary Material).

**TABLE 1:** Differential proteins of urine proteome.

Differential protein screening conditions	Number of differentially expressed proteins	Randomly generated number of different proteins	Differential protein reliability
FC ≥ 1.5 or≤0.67; P < 0.05	199	64.2	0.677
FC ≥ 2 or≤0.5; P < 0.01	51	6.6	0.871

### 3.3 Functional analysis of differentially expressed proteins

Differentially expressed proteins identified under strict screening conditions were selected for functional analysis. This analysis enriched a total of 44 biological processes and 7 signaling pathways (Supplementary Material), among which ten biological processes and two signaling pathways were related to the immune response (Table 2).

**TABLE 2:** GO functional enrichment analysis and KEGG pathway analysis of differentially expressed proteins.

Category	Term	Count	%	P-value
GOTERM_BP_DIRECT	Immune response-regulating signaling pathway	16	6.18	1.92E-15
	Immune response	23	8.88	1.30E-06
	Regulation of complement activation	3	1.16	3.17E-03
	Cellular defense response	5	1.93	5.73E-03
	Complement activation, alternative pathway	3	1.16	1.68E-02
	Complement activation, classical pathway	4	1.54	2.12E-02
	Immunoglobulin mediated immune response	5	1.93	4.49E-02
	Inflammatory response	11	4.25	4.70E-02
	Natural killer cell inhibitory signaling pathway	2	0.77	4.94E-02
	Natural killer cell activation	3	1.16	5.44E-02
KEGG_PATHWAY	Antigen processing and presentation	12	4.63	1.09E-07
	Complement and coagulation cascades	6	2.32	1.72E-02

### 3.4 Individualized analysis of urine proteome

**FIGURE 3:**
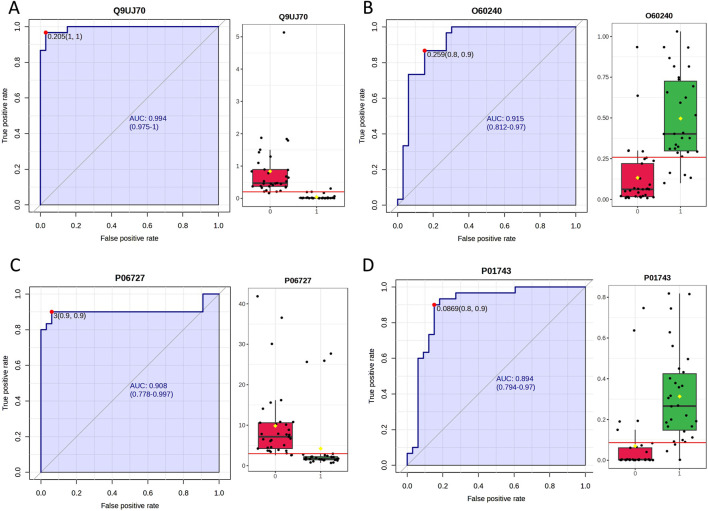
The top 4 AUC values of the significantly changed proteins by individualized analysis. **(A)** N-acetyl-D-glucosamine kinase (N-acetylglucosamine kinase). **(B)** Perilipin-1. **(C)** Apolipoprotein A-IV. **(D)** Immunoglobulin heavy variable 1–46.

Urinary proteomics provides a more comprehensive reflection of the body’s condition. To identify differentially expressed proteins with higher credibility, we focused on proteins showing consistent change trends among the significantly different proteins. We then calculated the corresponding AUC values for these proteins. To assess individual patient conditions, each sample was compared individually against 11 healthy controls. The differentially expressed proteins were shown in Table 3. Two differentially expressed proteins were identified in all 10 samples; five others were found in 9 TNs, all showing a relatively consistent expression change trend. The AUC values of these proteins ranged from 0.798 to 0.994, as summarized in Table 4; the top four AUC values were illustrated in Figure 3.

**TABLE 3:** Number of identified proteins in individualized comparison of urine proteomics under different screening conditions.

Screening parameter	Trend of differential protein change	The number of significantly different proteins in individuals									
		1	2	3	4	5	6	7	8	9	10
Adjusted p-value <0.05; FC1.5	Total	942	647	842	712	710	725	591	691	814	648
	↓	574	267	331	399	314	311	336	414	454	397
	↑	368	380	511	313	396	414	255	277	260	251
Adjusted p-value <0.01; FC2	Total	591	342	515	412	385	423	370	459	475	418
	↓	388	140	196	264	202	171	234	317	348	274
	↑	203	202	319	148	183	252	136	142	127	144

**TABLE 4:** List of proteins identified in urine by individualized analysis.

Protein ID	Protein name	Variation trend	Proportion	AUC value
Q9UJ70	N-acetyl-D-glucosamine kinase (N-acetylglucosamine kinase)	↓	9/10	0.994
P06727	Apolipoprotein A-IV	↓	9/10	0.908
Q86TX2	Acyl-coenzyme A thioesterase 1	↑	10/10	0.798
O60240	Perilipin-1	↑	10/10	0.915
Q8NE01	Metal transporter CNNM3	↑	9/10	0.851
Q9UHG0	Doublecortin domain-containing protein 2	↑	9/10	0.831
P01743	Immunoglobulin heavy variable 1–46	↑	9/10	0.894

## 4 Experiment discussion

### 4.1 Urine proteome could well demonstrate the characteristics of TN patients

This study was the first to analyze the urine proteome of patients with TN. There was no significant difference in gender and age between 11 healthy individuals and 10 patients with trigeminal neuralgia. The patients exhibited typical clinical manifestations of trigeminal neuralgia, which were alleviated by oral administration of carbamazepine. Some patients received nerve radiofrequency ablation; however, the treatment outcomes were unsatisfactory.

Visualization of the urine proteome from TN patients and healthy controls clearly distinguished different populations (Figure 1B). Most samples within the same group clustered together; however, some individuals showed similarity to samples from other groups. Because the specimen number was small, factors such as disease severity were not screened, which added complexity to the analysis. Nevertheless, this demonstrates that the urine proteome offers a unique advantage for individualized patient analysis.

### 4.2 Functional analysis of different proteins between groups

GO function analysis of biological processes revealed significant enrichment in immune response, natural killer cell activation, and natural killer cell inhibitory signaling pathways. KEGG analysis showed enrichment of antigen processing and presentation and complement-coagulation cascades, consistent with the enriched biological processes. These results indicate that immune-related responses detected in patients’ urine are closely associated with TN. The infiltration of inflammatory cells and the release of cytokines have been associated with pain (Balkowiec-Iskra, 2010; Iuodzhbalis and Sabalis, 1989; Duffy et al., 2019). In this study, we identified more than twenty immune-related proteins, including 13 types of killer cell immunoglobulin-like receptors (Supplementary Material). Natural killer cells are a type of lymphocytes and an important component of the immune system. Failure of their function can lead to various problems, including different types of cancers, infections, immunodeficiency syndromes, and even autoimmune diseases. Some natural killer cells can kill activated T lymphocytes that target the myelin surrounding nerve cells (Macchi and Mastino, 2016). Killer-cell immunoglobulin-like receptors (KIRs), expressed on the surface of NK cells, interact with HLA class I molecules on other nucleated human cells. This interaction results in immune tolerance when the targeted cells are healthy, and cytotoxic activity of NK cells against cancerous target cells. Multiple sclerosis, a peripheral nervous system disease, has been associated with KIRs (Shahsavar et al., 2016). In our study, both inhibitory and activating KIR types were detected. Based on this finding, we speculated that killer cell-related immune factors may play a crucial role in the onset and progression of trigeminal neuralgia. According to this mechanism, immune-related treatment of killer cells for patients with trigeminal neuralgia might be a worthy and potential treatment. Immune system pathways were associated with the therapeutic effect of microvascular decompression (Doshi et al., 2024), These response pathways were consistent with the urinary proteome, suggesting that the urinary proteome may serve as an effective and non-invasive method to monitor other treatments for TN.

### 4.3 Highly credible differential proteins in individual samples

A large number of significantly different proteins were found between the individual urine proteomes of TN patients and healthy controls. This number was much higher than that observed in the grouped protein analysis, further indicating that individual differences are widespread in real-world medical care. Urine proteomics theoretically reflects various changes throughout the body, making it challenging to identify biomarkers for a single disease. If proteins showing similar change patterns are identified among the numerous differentially expressed molecules, the correlation between these proteins and diseases would be strengthened. Therefore, identifying protein molecules with common characteristics through individualized analysis is more reliable. A total of seven high-confidence protein molecules were identified in this experiment, detected in more than eight individuals.

N-acetylglucosamine kinase was highly expressed and played a key role in the development of brain neuron dendrites (Lee et al., 2014; Islam et al., 2015). Apolipoprotein A-IV has been reported to significantly increase in perineural concentration in various animal models of sciatic nerve injury repair. The article speculates that it may participate in lipid transport in nerve tissue (Boyles et al., 1990). However, our study found that Apolipoprotein A-IV was significantly decreased in trigeminal neuralgia (TN), suggesting a weakened biological process in this condition. Doublecortin domain-containing protein 2 is widely distributed and highly expressed in the brain, where it inhibits the classical Wnt signaling pathway (Schueler et al., 2015). It is also associated with reading ability and the development of regulatory nerves (Meng et al., 2005). In our experiment, 9 out of 10 samples showed an increase, which likely relates to nerve damage.

In addition, Acyl-CoA thioesterase activity (ACOTs) mainly comes from a family of enzymes belonging to a broader group of thioester hydrolases (Hunt and Alexson, 2002). ACOT1 is a cytosolic enzyme selective for long chain saturated and monounsaturated acyl-CoAs. It modulates the cytosolic pool of these acyl-CoAs and FFAs, potentially controlling ligand availability for the nuclear hormone receptors PPARα and HNF4α (Dongol et al., 2007). Research showed that PPARα is associated with diabetic corneal neuropathy (Mansoor et al., 2024). The increase of ACOT1 in our experiment may establish a link with neurological diseases. Perilipin 1, an adipocyte-specific lipid droplet-associated protein, can inhibit the activation of the NF-κB inflammatory pathway and reduce the expression of tumor necrosis factor alpha (TNF-α), interleukin 1 beta, and interleukin 6 induced by lipopolysaccharide (Zhang et al., 2018). This further indicated that the immune response was involved in the onset of trigeminal neuralgia. CNNM3, a metal transporter, showed increased expression. This suggests that metal ion imbalance may be involved in disease development.

Meanwhile, we focused on some protein molecules that significantly changed in the grouped analysis. Discoidin, CUB and LCCL domain-containing 2 is a neurofibrin-like transmembrane scaffold receptor. It has known and expected roles in vascular remodeling and neuronal positioning, and is also upregulated in tumors (Schmoker et al., 2017). These high-confidence molecules are most likely to become disease-related markers.

### 4.4 Problems and prospects

However, although this experiment is the first attempt to use urinary proteomics technology to identify differences between TN patients and healthy individuals, there were still some deficiencies. One of the main problems was the small number of experimental specimens. P-value adjustment was used in the statistical analysis of data, to minimize generated deviations. Moderate or small differences may remain undetected, and estimation accuracy might be insufficient. Urine proteomics reflects the overall state of the body, not just diseases. Moreover, individualized analysis reveals significant differences among patients. At the same time, the small sample size made it impossible to stratify patients based on their clinical characteristics (disease course, severity, presence of other diseases, etc.). This may affect the clinical value of the differential proteins identified in the experiment. Therefore, the conclusions of this study should be considered preliminary and exploratory. Although promising trends have been observed, these findings need to be confirmed in future larger scale confirmatory studies. As this is an exploratory study, key differential proteins have not yet been independently validated by methods such as the Enzyme Linked Immunosorbent Assay (ELISA) and Western blot. Large-scale sample size or clinical cohort studies are needed in the future to clarify the application value of these key markers. The study identified a strong immune response in TN patients, particularly through the detection of 13 killer cell immunoglobulin-like receptors. However, current immune-related treatments for TN are limited, highlighting the need for further research.

## 5 Conclusion

This is the first study to comprehensively profile TN using urinary proteomics. It describes the common and differential characteristics of TN patients and provides important clues for disease pathogenesis and treatment. Some significantly different protein molecules have been reported for the first time in TN, offering broader clues for studying molecular functions. Furthermore, several disease-related proteins with greater significance were identified in the experiment, which may serve as markers to assist diagnosis and treatment.

## Data Availability

The original contributions presented in the study are publicly available. This data can be found here: The mass spectrometry proteomics data have been deposited to the ProteomeXchange Consortium (https://proteomecentral.proteomexchange.org) via the iProX partner repository with the dataset identifier PXD069864, The access link for data in iProX is: https://www.iprox.cn/page/project.html?id=IPX0013924000.

## Abbreviations

TN, Trigeminal neuralgia; LC-MS, liquid chromatography-mass spectrometry; FASP, filter-aided sample preparation; DDA, Data Dependent Acquisition; DIA, Data Independent Acquisition; FC, fold change; NAMPT, Extracellular Nicotinamide phosphoribosyltransferas.
